# The impact of working in COVID-19 hospital on indonesian nurses’ mental health and wellbeing: a qualitative study

**DOI:** 10.1186/s12912-022-01131-6

**Published:** 2022-12-07

**Authors:** Gregorius Abanit Asa, Nelsensius Klau Fauk, Melkianus Ratu, Paul Russell Ward

**Affiliations:** 1 Research Centre for Public Health, Equity and Human Flourishing (PHEHF), Torrens University Australia, Adelaide, South Australia Australia; 2 Sanggar Belajar Alternatif (SALT), Atambua, Nusa Tenggara Timur Indonesia; 3 Institute of Resource Governance and Social Change, Kupang, Indonesia; 4 Program Studi Keperawatan, Universitas Timor, Timor Tengah Utara, Nusa Tenggara Timur Indonesia

**Keywords:** Nurses, COVID-19, Mental health, Well-being, Resource poor settings, Indonesia

## Abstract

**Background:**

The outbreak of coronavirus disease (COVID-19) has impacted the lives of more than 580 million people and killed more than six million people globally. Nurses are one of the most impacted groups as they are at the frontline to fight against the virus and to try to save the lives of everyone affected. The present study aimed to explore the impact of working in COVID-19 wards on the mental health and wellbeing of nurses in the early stage of the pandemic in a hospital in East Nusa Tenggara, Indonesia.

**Methods:**

A qualitative study was conducted with 22 nurses, recruited using purposive sampling. Data collection was conducted from April to May 2022 and data analysis was guided by qualitative framework analysis.

**Results:**

The findings show that nurses experienced a range of mental health impacts including fear of being infected and infecting loved ones; fear of early death; psychological distress related to the conflict between the lack of personal protective equipment (PPE) and both professionalism and moral responsibility for patients; stress due to long waiting period to know the result of the COVID-19 tests; stress and worry due to public indifference and lack of role models; the negative impact of community doubt and distrust around COVID-19; and distress due to stigma and discrimination towards nurses caring for COVID-19 patients and their families.

**Conclusions:**

The current findings indicate further psychological intervention programs to support nurses, especially the ones in resource poor settings and enhance their psychological resilience.

## Introduction

Coronavirus disease 2019 (COVID-19) was first reported in December 2019 in Wuhan, China [1]. The virus spreads quickly and has resulted in a remarkable worldwide health emergency. Due to the wider impacts, the World Health Organization (WHO) declared COVID-19 as a worldwide pandemic in March 2020 [1]. By early August 2022, the disease had affected more than 580 million people and more than 6 million people died due to the virus [2]. In South-East Asia, COVID-19 has affected more than 55 million people [2].

Indonesia is one of the countries in the South-East Asia region where the number of confirmed cases keeps increasing. The first case was found on 2 March 2020 when two people were diagnosed with COVID-19 [3]. A month later, there were 4,557 cases of COVID-19 confirmed and 399 deaths [4]. Due to the massive impact, the Indonesian government announced COVID-19 as a national disaster on April 13, 2020 [5]. At the beginning of August 2022, the virus had affected more than 6 million people and more than 150,000 people died from the virus [6]. People working in the health sector or healthcare professionals (HCPs) in the country are one of the groups negatively affected by the pandemic. In April 2022, more than 2000 Indonesian health workers (representing 1.3% of the total death due to COVID-19 in Indonesia) including doctors, nurses, midwives, dentists, therapists, radiology assistants, medical laboratory assistants and ambulance drivers who had been vaccinated died due to COVID-19 infection [7]. The confirmed cases in the country are believed to have increased due to the government’s late response, lack of preparation to cope with the rapid spread of COVID-19, and insufficient health services, including a lack of hospital and Intensive Care Unit (ICU), limited trained medical and laboratory staff and the uneven access to health care across the archipelago [8, 9]. To prevent the further spread of COVID-19, the Indonesian government has established programs including prioritizing healthcare workers in the vaccination program, providing personal protective equipment (PPE), implementing community activity restrictions periodically, providing polymerase chain reaction (PCR) tests, and providing vitamins and supplements [10]. Lockdowns or Large-Scale Social Restrictions (known as Pembatasan Sosial Bersakal Besar/PSBB) and the Community Activities Restrictions Enforcement (known as Pemberlakuan Pembatasan Kegiatan Masyarakat/PPKM) were also imposed by the Indonesian government for some period between 2020 and 2022 to prevent massive transmission of the virus [11–13].

Globally, previous studies have reported that the increased number of COVID-19 cases caused mental health challenges for nurses working in COVID-19-designated hospitals. For example, nurses are reported to experience: fear of infection transmission to themselves and their families due to their prolonged exposure to the virus [14, 15]; stress, anxiety and depression [16, 17] due to their involvement in healthcare service delivery to COVID-19 patients; and feeling insecure due to a lack of precautionary measures such as Personal Protective Equipment (PPE) [18]. The increasing number of confirmed cases and death, lack of specific treatment, and having to stay in quarantine are also contributing factors to the mental health challenges they face. Similarly, stigma, distancing behavior, discrimination [19] and feeling isolated in the face of health threats and high-intensity work [20] leading them to postpone eating or drinking to avoid toilet breaks to care for patient loads [14] are factors affecting nurses’ mental health.

Although a range of impacts of COVID-19 on health workers have been well documented in many settings, evidence on the mental health impacts nurses face during caring for patients with COVID-19 in resource poor settings, including Indonesia, is still limited. Several recent systematic reviews have highlighted the impacts of COVID-19 on HCPs covering only a few resource poor countries, with the majority conducted in China [21, 22]. In addition, there is limited evidence of in-depth qualitative exploration of the impact of working in the COVID-19 hospital on nurses’ mental health and wellbeing. This study aims to fill in these gaps in knowledge by exploring how nurses were impacted during the early stages of the pandemic in a hospital in Belu district, a resource poor setting in Indonesia – this was the only hospital where patients with COVID-19 were treated during the early stages of the epidemic in Belu. Understanding how COVID-19 impacted the mental health and wellbeing of nurses is critically important as it can help in the development of policy and practice to protect their mental health and wellbeing.

## Methods

Our study employed a qualitative, inductive approach since we were interested in the world-views of the participants and were keen to limit our preconceptions of the impact of working during the COVID-19 pandemic on nurses’ mental health. In terms of method, we used semi-structured interviews, which allowed for explorations and discussions of relevant experiences and perceptions of working in a hospital during the pandemic, in addition to creating an atmosphere conducive to an open and uninhibited flow of conversation. The interviews were therefore considered to be a social encounter in which knowledge was constructed and not simply an occasion for information gathering. In this way, the interview process allowed the space for participants to reflect on their experiences before and during the pandemic, to allow them and us to interpret the factors influencing their motivations and behaviours and ultimately the impact on their mental health and wellbeing.

### Study setting

The study was conducted in Belu district, East Nusa Tenggara Province, Indonesia. In regards to the COVID-19 pandemic, as of 1^st^ August 2022, there have been 6,207,098 confirmed COVID-19 cases in Indonesia. Of the total number, 6,001,402 people have fully recovered, 156,993 people died and 48,703 people are being treated [2]. In East Nusa Tenggara, where Belu district is located, the current data as of 1^st^ August 2022 report a total of 94,165 confirmed cases, of which 92,534 people have fully recovered, 1,525 people died and 106 people are being treated [23]. The district has a total population of 204,541 people that are distributed in 12 sub-districts [24]. The district is located in the Eastern part of the province and directly shares the border with Timor-Leste. It has one public hospital where patients with COVID-19 were quarantined during the early stages of the COVID-19 epidemic, one army hospital, and two private hospitals.

### Data collection

Data collection was conducted in April and May 2022. Due to the COVID-19 protocols, it was conducted using phone and zoom interviews. Participants were nurses aged over 18 years and providing health care services for COVID-19 patients at the hospital where COVID-19 patients were treated. Participants were recruited using a purposive sampling method, recruiting only nurses who worked on COVID-19 wards with patients diagnosed with COVID-19. Copies of the study information sheets containing the contact information of the field researchers (GAA, MR) were posted on the information board and made available at the front desk of the hospital. Participants who called and texted to confirm their participation were recruited for an interview at a researcher-participant mutually agreed upon venue and time. Finally, 22 nurses participated in the study.

Interviews were focused on key areas, including nurses’ views and experiences regarding their mental health and the impact of working in a COVID-19 hospital with patients with COVID-19. The interviews were guided by several predetermined main questions and probing questions were developed during the interview [25]. Some examples of the main questions are “How do you feel about treating patients with COVID-19? What do you fear the most when having close contact with patients with COVID-19? What mental health challenges have you experienced when treating patients with COVID-19? What do you think about COVID-19-related PPE for HCPs in the hospital? What do you think about people’s adherence to COVID-19 prevention protocols? What is your experience about people’s attitudes and behaviours towards you and your family members, considering their knowledge of your close contact with patients with COVID-19?” The decision about the questions was made through a process of formulation, discussion and revision. The interviews were carried out in Indonesian, the primary language of both participants and researchers. Each interview lasted between 40 to 60 min and was digitally audio recorded. Before commencing the interviews, the researcher explained the objectives of the study and provided an opportunity for participants to raise questions or ask for clarification. There was no established relationship between the researchers and any of the participants before the interview. Participants were advised that they could withdraw at the beginning, during and/or after the interview. Participants were allowed to pause or change the topic during the interviews whenever they felt uncomfortable talking about anything. The study was approved by the Health Research Ethics Committee, Duta Wacana Christian University, Yogyakarta, Indonesia (Ref. No.: 1380/C.16/FK/2022).

### Data analysis

Before commencing the analysis, the audio recordings were transcribed verbatim into coding sheets by the researchers (GAA, MR). The process of data analysis was both deductive and inductive and followed methods described by Ritchie and Spencer’s five steps framework analysis [26, 27]. The five steps include (i) *familiarization* by reading each transcript repeatedly, marking ideas and providing comments to the data extracts to search for meaning, pattern and ideas; (ii) *identifying a thematic framework* by making judgements and writing down key issues and concepts from the participants; (iii) *indexing all the data* by creating open coding to look for similar or redundant codes and reduce them into smaller number by grouping together to reach a few overarching themes and sub-themes; (iv) *creating a chart* by arranging thematic framework so that data could be compared within each interview and across all interview; (v) *mapping and interpretation* data as a whole [26, 28]. Data analysis was performed in Indonesian and then the selected quotes for publication were translated into English by authors (GAA, NKF) who speak both languages. The translation was then checked for accuracy and clarity by other authors (MR, PRW).

## Results

A total of 22 nurses were interviewed, consisting of 14 females and 8 males. All participants had an educational background in nursing either diploma III or undergraduate and worked at the hospital where COVID-19 patients were treated. All participants were involved in healthcare service delivery to COVID-19 patients. The details of the participants are presented in Table 1.

**Table 1:** Characteristics of the participants

Respondent No	Age, years	Gender	Work experience, years
R1	27	Female	5
R2	32	Female	7
R3	37	Male	8
R4	28	Female	3
R5	30	Female	7
R6	34	Male	8
R7	35	Male	6
R8	29	Female	4
R9	41	Female	13
R10	39	Female	12
R11	33	Male	7
R12	27	Male	4
R13	29	Female	3
R14	36	Female	12
R15	36	Female	8
R16	29	Female	4
R17	33	Male	7
R18	28	Female	4
R19	30	Female	5
R20	45	Female	15
R21	34	Male	7
R22	37	Male	9

**Fig. 1:**
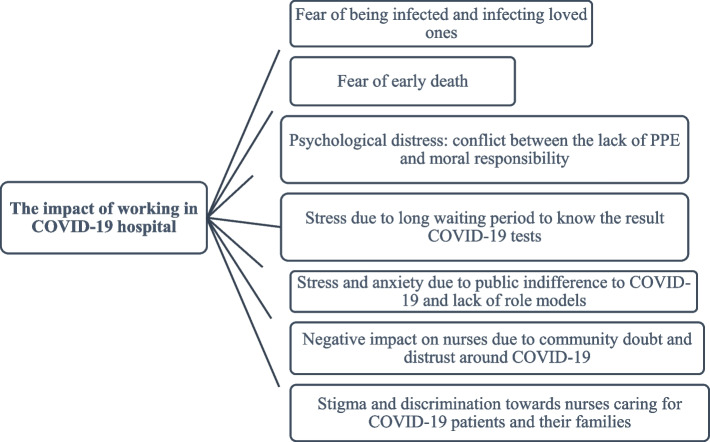
Themes extracted from the data

The findings were categorized into several themes (see Fig. 1) which are used to structure this section of the paper.

### Fear of being infected and infecting loved ones

The increased number of people and healthcare workers infected with COVID-19 seemed to have put nurses under unprecedented strain. Participants described experiencing fear of being infected and transmitting the virus to their loved ones. Such fear was influenced by their awareness of the quick and easy spread of the virus and the close contact they had with COVID-19 patients due to the nature of their work. Participants’ understanding of not being fully protected by the PPE they used and their awareness of failing to follow the instructions to properly use PPE were also factors that contributed to the heightened fear of contracting the virus. They also felt that they needed to hide their fear from their families, in an attempt to protect them. The following quotes described such experiences:

*“I already feared being infected when there were no COVID-19 patients in the hospital and I felt more scared when the number of patients with COVID-19 in the hospital increased. I thought, it is just a matter of time being infected because the virus spreads so quickly and I am a nurse caring for COVID-19 patients. But you know, I did not want to show my fear to my family. I am scared, if I am infected then I may spread it to my family members” (R2: 32 years old).*

The narratives of other nurses also show that even though they talked about the availability of PPE and appropriate procedures to wear and take off PPE, they admitted to not always following the procedures. This increased their chances of getting infected and spreading the virus to others, including their family members, as illustrated in the following story:

*“I am a nurse and there is no guarantee I will not be infected although I wear gloves, facemask and apron. You know, when we (nurses) deal with a highly contagious disease, we need to wear facemasks, gloves, aprons and face shields correctly and properly. It is the same when we take off all the stuff. We need to remove gloves first, gown and then masks. However, in practice, I tended to forget the sequence. Some also may not understand the infection control procedure leading them to easily get infected” (R9: 41 years old).*

Similarly, their stories showed that their understanding of asymptomatic carriers was also a contributor to their fear of contracting the virus, which made them often feel worried and suspicious about the possibility of contracting the virus. The next quote highlights the constant worry that nurses felt because asymptomatic people with COVID-19 often “felt or looked healthy” and as such, were less likely to wear facemasks. For this nurse, that increased her worry because the risk of being infected and transmitting to her family was higher:

*“What makes more scared is people without COVID-19 symptoms who could spread the virus. We might think they are healthy because they do not have any symptoms like coughing or fever. Because they feel or look healthy, they are less likely to be wearing facemasks or quarantining. So, I could get the virus from them and I may transmit it to my parents and other family members. I often feel worried and suspicious about myself contracting the virus every time I feel tired or a bit unwell” (R15: 36 years old).*

The participants’ sub-optimal home living situations seemed to also exacerbate their fear and worry about the possibility of the virus spreading among their family members. The lack of rooms at home for proper quarantine after interacting or having close contact with COVID-19 patients in the hospital led them to increase the chance in being in close contact with other family members. Nurses also talked about sharing a variety of household utensils and equipment, increasing the chances of viral transmission. Similarly, those renting a room in shared house had to share a bathroom and toilet with other tenants, which not only caused inconvenience to other tenants during self-quarantine but also increased the fear and anxiety of transmission of COVID-19. Such experiences are reflected by the following statements:

*“I quarantined myself at home when one of the nurses tested positive. My home is small with three bedrooms and six people were living in the house. Normally, we shared rooms. But when I was in quarantine, I used one bedroom and the others had to share the two bedrooms remaining. In the evening, some slept with me in the living room, I was so worried. So, you know, I was in quarantine but still had contact with other family members and shared a bathroom, toilet and corridor. It was a very stressful situation…” (R11: 33 years old).*

*“It was a bit hard for those who lived in kost (renting a room). You know, not all kost have a bathroom directly connected to the bedroom. Toilet and bathroom are built separately from the bedroom. This means, they have to share bathroom and toilet with other tenants, and other tenants might not be happy” (R12: 27 years old).*

### Fear of early death

Personal knowledge and stories in the media about the death of young people, including nurses and doctors, due to COVID-19 were reported as supporting factors for the excessive fear of early death among participants. A few participants commented that they were still young and having the responsibility for families, including raising and preparing the future of their children, were also factors that exacerbated their fear of early death. Some nurses talked about planning to start a family and others had only recently embarked on careers in nursing. The following narrative links the fear of early death to their role as a parent of young children, and also to the fact that doctors and nurses were dying in “reputable hospitals” even though they were wearing PPE. These increased their perceived fear of death from COVID-19:

*“I felt anxious and a bit stressed any time I read news on Facebook about young nurses and doctors who worked in national and reputable hospitals dying due to COVID-19 infection although they wore gloves, facemasks and aprons. That is really scary. You know, I do not want to die early because I am still young and I have kids. They really need me and, of course, I have the responsibilities for their future” (R 10: 39 years old).*

In the next quote, the nurse made specific reference to young nurses dying of COVID-19, which made it feel “close”. This particular nurse is also young and was in the process of planning a wedding, and the fear that ensued impacted her mental health:

*“Knowing young people died due to COVID-19, I feel like death is so close to me. You know, I am not married yet and I plan to get married next year. I pray to God to protect me from the virus. To be honest, sometimes, the fear of early death affects me” (R5: 30 years old).*

The fear of early death and the potential impact on their lives and the lives of others (e.g. their young children, their future spouse, etc.) had led some of our participants to consider switching jobs (to move out of nursing completely) or moving to other departments within the hospital that had lower risks of COVID-19 infection. However, the nature of their particular nursing qualification meant that moving to other parts of the hospital was difficult because they did not have the appropriate specialization. In addition, the unavailability of other job opportunities outside of nursing made it very difficult to have a career change, partly due to the economic implications of the pandemic due to lockdowns and shrinking of many industries within Indonesia. Such concerns were described by several nurses as follows:

*“I am a civil servant nurse (a nurse who works for the government. She is a government employee and can only work for the government hospital or community health centre or health department). I never felt scared like today because of this deadly virus. If there is a chance to move to other departments other than health or hospital, I would take it because I do not want to risk myself and my family. It is hard to move to other departments such as education and tourism because my educational background is nurse or health only” (R6: 34 years old).*

*“I am a casual worker nurse in the hospital. I discussed with my husband about finding another job that has low risks of getting infected with the deadly virus. However, you know, in this current situation it is hard to get another job in this district” (R1: 27 years old).*

### Psychological distress: conflicts between the lack of PPE and moral responsibilities

Lack of adequate PPE in the hospital where the participants worked was reported as a contributing factor to their mental health and stress. This seemed to also encourage their decision not to engage in nursing work under conditions where PPE was unavailable, which was considered a strategy to avoid putting their lives at risk. Such an action was supported by the perception that the availability of PPE and other safety equipment to support healthcare service delivery to COVID-19 patients was the responsibility of the healthcare facilities (the hospital). Difficulties in obtaining facemasks and the sudden increase in price of facemasks in stores in Belu were also reported as factors affecting their mental health. Quotes below described such experiences:

*“When one or two patients with COVID-19 were quarantined in the hospital, we (nurses) were panicked and stressed. We (nurses) went to the hospital but we did not want to work because of a lack of adequate facemasks, gloves and aprons. It was kind of our protest. It was covered in the news. You know, we did not want to risk our life” (R11: 33 years old).*

*“It was hard to find facemasks in pharmacies or stores at the beginning of the COVID-19 case here (Belu). If there was, the price was more expensive than before the pandemic. This was a very stressful situation because I think the hospital is responsible for providing facemasks for us” (R7: 35 years old).*

However, refusing to work due to the lack of PPE was acknowledged as putting the participants in a difficult situation which created a dilemma due to their awareness of negative health consequences for COVID-19 patients and their moral obligation to patient safety. On the one hand, patient safety is part of a nurse’s duty of care, but on the other hand, nurses need to care about their own health and have embodied obligations to their families. This difficult and almost intractable situation for nurses impacted their mental health and their sense of self – for the first time, nurses in our study were forced to choose between the safety of their patients (i.e. the reason why they went into nursing), their own safety and the safety of their families. The COVID-19 pandemic had caused psychological distress for nurses, who felt the need to make difficult choices in short periods of time, with little moral or professional guidance. The conflict between professional and moral responsibilities and personal safety was described by participants as follows:

*“My friends and I refused to work because there were no facemasks and aprons. However, you know, we were in dilemma. We could not let patients stay in the hospital without care. They (patients) might wonder why nurses did not visit or provide them with medicines. They might die easily” (R10: 39 years old).*

*“It was not easy. As a nurse, I have a commitment to providing care for patients. I refused to work due to the lack of PPE, but my heart went to the patients. I refused because I have kids and family who rely on me” (R14: 36 years old).*

### Stress due to long waiting period to know result of the COVID-19 tests

A few participants acknowledged that the long waiting period to know the result of the COVID-19 tests contributed to their stress. This was due to the lack of a COVID-19 swab test laboratory services in the district. As the consequence, all samples were sent to the provincial laboratory, which took 2–3 weeks to provide results. This long waiting period was also reported to provide space for additional spread of the virus in the community. Participants provided examples of patients being impatient whilst waiting for their results, assuming they were negative (until they received results) and concomitantly engaging with the community. Nurses suggested that the lack of local laboratory services and the resulting long waiting times may have increases COVID-19 community transmission. The following quotes illustrate these points:

*“The hospital did not have a swab laboratory. All the samples have to be sent to Kupang (provincial laboratory), and the results are informed in two or three weeks. You know, patients will keep asking about the result. We (nurses) always said to them to just wait but we do not know how long they would be patient enough to wait” (R2: 32 years old).*

*“It takes time to know the result of the swab test. Some patients who are quarantined at home are impatient and they might have engaged with other family members in a normal situation because they think they are okay or healthy or have no symptoms anymore. This situation makes us stressed and worried about the virus spread in the community” (R8: 29 years old).*

### Stress and anxiety due to public indifference to COVID-19 and lack of role models

Some participants mentioned a lack of public awareness of the risks of COVID-19 and even a sense of public indifference, reflected in other people hosting and attending non-COVID-adjusted parties. These were described to be out of their control, but nonetheless made them worried and stressed during the pandemic due to the awareness of the possibility of rapid community transmission of the infection through such events:

*“We have a very limited number of medical doctors and lack health infrastructures and COVID test tools. But you know, there are still wedding parties and many people attend those parties. They gather, chat and dance together. They are not scared of the increasing number of infected patients and deaths due to the virus. Some do not care about the warnings. These kinds of events make me worried and stressed” (R9: 41 years old).*

*“I think those hosting wedding parties and guests attending the parties are not afraid of the disease or they might not care at all. My friends and I work in the hospital and struggle to look after infected patients, we are worried because the number of patients with COVID increases every day” (R4: 28 years old).*

In addition to public nonadherence related to social events, political events were also reported to contribute to nurse’s stress and worry. Participants mentioned that they were worried because crowds gathered without wearing facemasks, standing close to others and shouting to support political leaders during political campaigns. Such nonadherence was acknowledged to be supported by a lack of role models from local leaders and politicians who gathered crowds without considering COVID-19 protocols and a lack of health resources in the district. The following quotations describe how such challenges occurred and caused mental health challenges to nurses:

*“Many people did not care about COVID-19 protocols during political campaigns. In the crowd, they just gathered, shouted, sang, stood close to each other and hugged. You know, they did not care about health and death, but we (nurses) were really worried and sometimes felt stressed” (R4: 28 years old).*

*“Sometimes I felt stressed with political campaigns. They (politicians) campaigned about people’s health and asked people to stay healthy. But you know, they (politicians) continued to gather lots of people during their campaigns and they did not provide facemasks” (R15: 36 years old).*

### Negative impact on nurses due to community doubt and distrust around COVID-19

Nurses provided numerous examples of patients, patients’ families, and indeed their own families questioning whether people were’really’ contracting and dying of COVID-19. This needs to be set within the context of post-Trump ‘Fake news’ and the huge social media presence of conspiracy theories and misinformation about COVID-19. Nurses fully accepted that family members would and should be entitled to question them about the death of their relatives – that was part of the role of being a nurse. However, nurses acknowledged feeling overwhelmed and negatively impacted when family members and friends consistently questioned the diagnosis of patients with COVID-19 and doubted COVID-19 as the cause of the death of patients who died in hospital quarantine. Nurses in our study assumed that such questions and doubt stemmed from community assumptions that healthcare professionals and hospitals too easily blamed COVID-19 as the main reason for a patient’s death and used it as a controlling strategy to make people adhere to COVID-19 protocols. Such feelings were explained by several participants:

*“My friends texted me asking whether the patients died due to COVID-19 or due to other diseases. I explained that the patients had COVID-19 symptoms and had been quarantined in the hospital for several days. But, again, they still doubted it. You know, sometimes I felt annoyed by their questions. They should have known that many people around the world died due to this deadly virus” (R13: 29 years old).*

*“Some did not believe that their family members died because of COVID-19. They thought that the hospitals just made up the reason so their family members became afraid and could not come in large numbers to mourn in the hospital. I really felt annoyed with this assumption. People thought that this was a strategy from the hospital so that people do not ignore the COVID-19 protocols” (R14: 36 years old).*

These quotes highlight that nurses perceive a level of community distrust in healthcare professionals and the hospital system regarding the management and treatment of COVID-19.

### Stigma and discrimination towards nurses caring for COVID-19 patients and their families

The stigmatising attitude of community members against nurses working in the hospital was reported as a contributing factor to nurses stress and anxiety. The suspicion towards these nurses as the carriers of COVID-19 in the community due to their close contact with COVID-19 patients they treated was the main reason for the stigma against them by other community members. This was reflected nurses talking in interviews about the dissemination of information within the community to avoid healthcare professionals working in the hospital. This also caused difficulty for them to convince people about their negative status of COVID-19 as reflected in the following narratives:

*“There was a message spread in the community (mouth to mouth news) to not to be close to nurses or healthcare workers because we (nurses) might bring the virus and would infect people. That made me a bit stressed and anxious at the beginning of the COVID-19 pandemic. This happened when some patients with COVID-19 were quarantined in the hospital (R13: 29 years old).*

*“After several days being quarantined at home, I tested and the result was negative. But you know, it was hard to convince people. People thought that I still had corona because I work in hospital” (R2: 32 years old).*

Being socially excluded was reported as a miserable experience. Participants reported losing valuable social experiences such as chatting and incidental social interactions with families, neighbours or other community members. This was reflected in the avoidance of physical contact with them due to the suspicion of their COVID-19 status and the fear of transmission:

*“I was in quarantine because I had a fever and runny nose. But I was grateful because the result of the COVID-19 test was negative. However, you know, my relatives kept their distance from me when they visited my home. They stood away from me. They did not chat with me. I felt a bit excluded but then I realized it was OK because they did not want to get infected or they followed the COVID-19 protocol. I didn’t like and sometimes felt angry and stressed because of their attitudes and behaviours” (R6: 34 years old).*

*“Some family members and neighbours were afraid to sit next to me even though there was no COVID-19 patient in the hospital and we finished quarantine and tested negative. I knew it was good during the pandemic but sometimes it is uncomfortable and stressed me out. It seemed as if people did not like us” (R15: 36 years old).*

Furthermore, as echoed by some participants, stigma attributed to nurses’ family members, reflected in labeling nurses’ whole families as COVID-19 carriers, was also reported as a contributing factor for stress and worry among the nurses. Participants described how their work in delivering COVID-19-related care and treatment for patients led to suspicion towards their families as virus carriers in the community which made them more stressed as elucidated in the following statement:

*“My neighbours suspected my family because I worked in hospital and had close contact with COVID-19 patients. So you know, I also felt a bit stressed with the stigma against my whole family” (R14: 36 years old)*

## Discussion

This study explored the impact of working in the COVID-19 designated hospital on the mental health and wellbeing of nurses in Belu District, Indonesia. Consistent with previous research [14, 15, 29], the current study highlights the immense impacts of the COVID-19 pandemic on nurses’ mental health, reflected in the fear of being infected and infecting their loved ones. The nature of their work, their awareness of failing to follow the procedures of donning and doffing PPE properly, and knowing being carriers of the disease without any symptoms, were factors that significantly contributed to the nurse’s stress and anxiety. All of these findings are in line with findings from previous studies [16, 30]. The study also found that although home quarantine was recommended as an important strategy to prevent community transmission of COVID-19 [31, 32], it had problems in resource poor settings, reflected in quarantine difficulties both for those who lived in small homes with limited bedrooms and those who rent a room for living. Room limitation led participants to experience sub-optimal quarantined conditions at home, a condition allowing them to easily have contact or share household items with other family members, leading nurses to feel more worried, anxious and stressed due to close exposure to infection.

As reported in previous studies [33, 34], the current findings also indicate that poor economic conditions have a significant influence on the efforts of community members and HCPs to comply with preventive protocols and prevent further community transmission of COVID-19. Being at high risk for COVID-19 infection was also reported to exacerbate nurses’ fear of early death. Such fear was also influenced by the news of an increased number of death of young HCPs (nurses and doctors) due to the virus contracted through healthcare-related close contact with patients with COVID-19, which conform with other studies [35, 36], leading nurses to distance or hinder themselves from caring for patients to the best of their abilities. As a consequence, some nurses considered switching or leaving their job, although at the same time they were unsure which jobs they would take as there were not many jobs available during the outbreak, as found in other studies [20, 37].

Consistent with previous studies [18, 38], the findings of the study suggest that nurses experienced a detrimental impact physically and mentally due to a lack of PPE and health resources. Such inadequate availability of PPE raised ethical dilemmas about the obligation to work, safety concerns, and the extent that nurses can provide optimal care to patients with COVID-19. If nurses did not perform their professional skills in the hospital, they might be deemed neglecting patients, which could result in death due to lack of care. Similarly, a long waiting period (2–3 weeks) to know the result of swab test samples due to the unavailability of a swab test laboratory in the study setting was reported as contributing to nurses worry and stress. Such a waiting period was considered not effective to prevent the spread of the virus due to patient’s impatience to know the result and to engage with family or community. The current findings also reflect a bigger picture of the unpreparedness of the healthcare system in the study setting to respond to the pandemic, which seems to negatively affect the work of HCPs and be a part of slow response during the early stages of the pandemic [39, 40]. The findings indicate the need for the provision of adequate laboratories in all hospitals in East Nusa Tenggara and other parts of Indonesia to face the current and future outbreaks.

The study suggests that family member’s and friend’s skepticism about the ‘real’ diagnosis of patients with COVID-19 or death status due to COVID-19, in addition to suspicion that doctors or nurses tended to “make up” the reason or “blame” COVID-19 as the cause of death to make people adhere to the prevention protocols, were also implicated in causing mental health challenges for nurses. Such skepticism can likely influence people’s attitudes and behaviors towards COVID-19 prevention protocols (distrust) proposed by the government, health authorities and healthcare professionals [33, 41]. This skepticism seems to be supported by the misinformation about COVID-19, distrust and public trust in conspiracy theories, which may affect people attitudes and behaviors to follow COVID-19 protocol and access to healthcare services during the pandemic and adherence to COVID-19 protocols as reported in previous studies [33, 42].

Consistent with other studies [19, 20] the findings of the current study suggest that lack of public awareness, public indifference, lack of role models, stigma and discrimination significantly affected nurse’s mental health and wellbeing. Political campaigns and social events (wedding parties) where people gathered and ignored COVID-19 protocols were reported to act as drivers for public disobedience. Such events seemed to highlight the absence of role models from government and politicians as well as the lack of public awareness to halt the spread of the contagious virus within the community. Thus, it can be argued, that the public tended to see the outbreak not as a serious concern. Similarly, the current findings underline the stigma against both the nurses and their family members as virus carriers within the community, which led to discriminatory behaviours towards them by families, neighbours and other community members. These findings support other studies that health care workers experienced distancing behaviors and discrimination from families and the public [19, 43], which then increased nurses psychological distress. Evidence from Canada shows that more than 30% of the general population did not want to be close to healthcare professionals, particularly those working in hospitals, as they were suspected of being COVID-19 carriers [19]. As a result, nurses experienced the miserable feeling of being socially excluded, reflected in limited social interaction and being treated differently, which in turn is highly likely to cause negative mental health challenges to them and their family members.

## Limitations and strengths of the study

The study has several limitations that need to be considered when interpreting the findings. It involved a relatively small number of participants; thus, the findings simply reflect the views and experiences of nurses in this particular setting, which might be different from those of nurses in different settings. Phone call interviews were also a limitation as these led to the inability of the researchers to observe the facial expressions and body language of interviewees, which may be useful for the analysis and interpretation of participant’s experiences. However, to the best of our knowledge, the study was the first qualitative inquiry that explored mental health and wellbeing experienced by HCPs delivering COVID-19 health care services to patients in East Nusa Tenggara and Indonesia. Thus, the findings are useful to inform the governments at local and national levels to provide the support required by HCPs who are the frontline workers in the fight against the COVID-19 pandemic.

## Conclusions

The study reports on the mental health impacts experienced by nurses working in a COVID-19 hospital during the early stages of the pandemic in Belu, Indonesia. The key findings of the study are: nurses experienced fear of being infected and infecting loved ones; fear of early death; psychological distress related to conflict between the lack of PPE and professional and moral responsibility for patients; stress due to long waiting period to know the result of the COVID-19 tests; stress and worry due to public indifference and lack of role models; negative impact of community doubt and distrust around COVID-19; and distress due to stigma and discrimination towards nurses caring for COVID-19 patients and their families. The current findings indicate the need for psychological intervention programs to support nurses, especially the ones in resource poor settings and enhance their psychological resilience. Future studies that explore challenges faced by family members of HCPs providing COVID-19 health care services to patients are recommended. As our findings indicated the intention of nurses to switch jobs due to COVID-related burden, a longitudinal study to explore how many healthcare professionals changed their jobs during the COVID-19 pandemic is also recommended.

## Data Availability

Data used in the study are available from corresponding author upon reasonable request.

## Abbreviations

COVID-19: Coronavirus disease 2019
PPE: Personal Protective Equipment
WHO: World Health Organization
HCPs: Healthcare Professionals
ICU: Intensive Care Unit
