# Correction: The impact of working in COVID-19 hospital on Indonesian nurses’ mental health and wellbeing: a qualitative study

**DOI:** 10.1186/s12912-022-01155-y

**Published:** 2022-12-27

**Authors:** Gregorius Abanit Asa, Nelsensius Klau Fauk, Melkianus Ratu, Paul Russell Ward

**Affiliations:** 1 Research Centre for Public Health, Equity and Human Flourishing (PHEHF), Torrens University Australia, Adelaide, South Australia Australia; 2 Sanggar Belajar Alternatif (SALT), Atambua, Nusa Tenggara Timur, Indonesia; 3 Institute of Resource Governance and Social Change, Kupang, Indonesia; 4 Program Studi Keperawatan, Universitas Timor, Timor Tengah Utara, Nusa Tenggara Timur, Indonesia

**Correction: BMC Nurs 21, 345 (2022)**

**https://doi.org/10.1186/s12912-022-01131-6**

Following publication of the original article [1], the authors reported an error in the authorship. The correct authorship has been updated in this Correction.

The original article [1] has been corrected.

## References

[1] Asa GA, Fauk NK, Ratu M (2022). The impact of working in COVID-19 hospital on indonesian nurses’ mental health and wellbeing: a qualitative study. BMC Nurs.

